# “We need all hands on deck”: characterizing addiction medicine training in Canada—a mixed methods study of fellowship program directors

**DOI:** 10.1186/s13722-025-00543-4

**Published:** 2025-02-19

**Authors:** Clara Lu, Kathryn Chan, Leslie Martin, Nadia Fairbairn

**Affiliations:** 1 Department of Medicine, McMaster University, Hamilton, ON Canada; 2 British Columbia Centre on Substance Use, Vancouver, BC Canada; 3 Department of Medicine, University of Ottawa, Ottawa, ON Canada; 4 Department of Medicine, University of Toronto, Toronto, ON Canada; 5 Department of Medicine, University of British Columbia, Vancouver, BC Canada

## Abstract

**Background:**

Addiction Medicine training in Canada has evolved substantially in the last few years with the establishment of accreditation standards and several new fellowship programs. The novelty of these formal training programs, created in response to complex and ever-expanding clinical needs in Addiction Medicine, creates unique educational circumstances that must be understood to support future growth. This study characterizes the current state of these postgraduate training programs in Canada through the perspectives of Program Directors (PDs).

**Methods:**

This study is a mixed methods study of 12 PDs. In Phase 1, participants completed a quantitative survey analyzed through descriptive statistics. In Phase 2, participants underwent a qualitative semi-structured interview that was coded with a thematic analysis approach. Mixing occurred both during the interim analysis between phases and during the interpretation stage.

**Results:**

28 trainees enrolled in a fellowship program in 2021–22 across 10 programs, and 27 trainees enrolled in 2022–23 across 11 programs. In each year, there were significantly fewer available spots than applications (31% and 29%, respectively). PDs identified a funding “bottleneck” as the most difficult and important challenge facing programs, with trainees supported by diverse and unstable funding sources. Qualitative analysis highlighted the need for sustainable funding models, flexibility toward alternative training pathways (shorter durations of training and re-entry from practice), and establishment of a national community of practice to support the co-creation of a robust addictions medical education infrastructure.

**Conclusion:**

For Addiction Medicine training to meet workforce demands, PDs stressed that funding was the challenge of prime importance. Future studies should examine the perspectives of Addiction Medicine fellows, the clinical and research impacts of fellowship graduates, and the cost-effectiveness of fellowship funding models.

**Supplementary Information:**

The online version contains supplementary material available at 10.1186/s13722-025-00543-4.

## Background

In Canada, over 200,000 deaths were attributable to substance use between 2015 and 2017 [1]. The opioid crisis accelerated these losses, amassing over 38,000 deaths due to acute opioid toxicity since 2016 [2]; strikingly, Statistics Canada reported in 2017 that life expectancy did not increase for the first time in over 40 years, a phenomenon “largely attributable to the opioid crisis” [3]. In 2021, hospitalizations entirely attributable to alcohol (277 per 100,000) surpassed hospitalizations for acute coronary syndrome (214 per 100,000) in Canada [4]. The COVID-19 pandemic worsened substance-related harms by exacerbating mental illness and social isolation, reducing access to harm reduction services, and creating a more dangerous drug supply [5]. The overall cost of substance use in Canada in 2020 was estimated at $49.1 billion [6].

Despite these immense harms and costs, Addiction Medicine has received minimal attention within medical curricula, reflecting a lack of prioritization, coordination, and standardization within medical education at local and global levels [7–11]. This has been characterized as a “monumental missed opportunity” [12] for both undergraduate and postgraduate medical education [13, 14]. Often viewed as a purely elective experience, addictions training in Canada has historically been available mainly to Family Medicine and Psychiatry trainees [7, 10, 15]. Calls to re-centre Addiction Medicine as a core skill have recommended a coordinated approach to medical education, aiming for both competence among generalists and expertise among specialists in Addiction Medicine [7, 8, 10, 16].

**Fig. 1:**
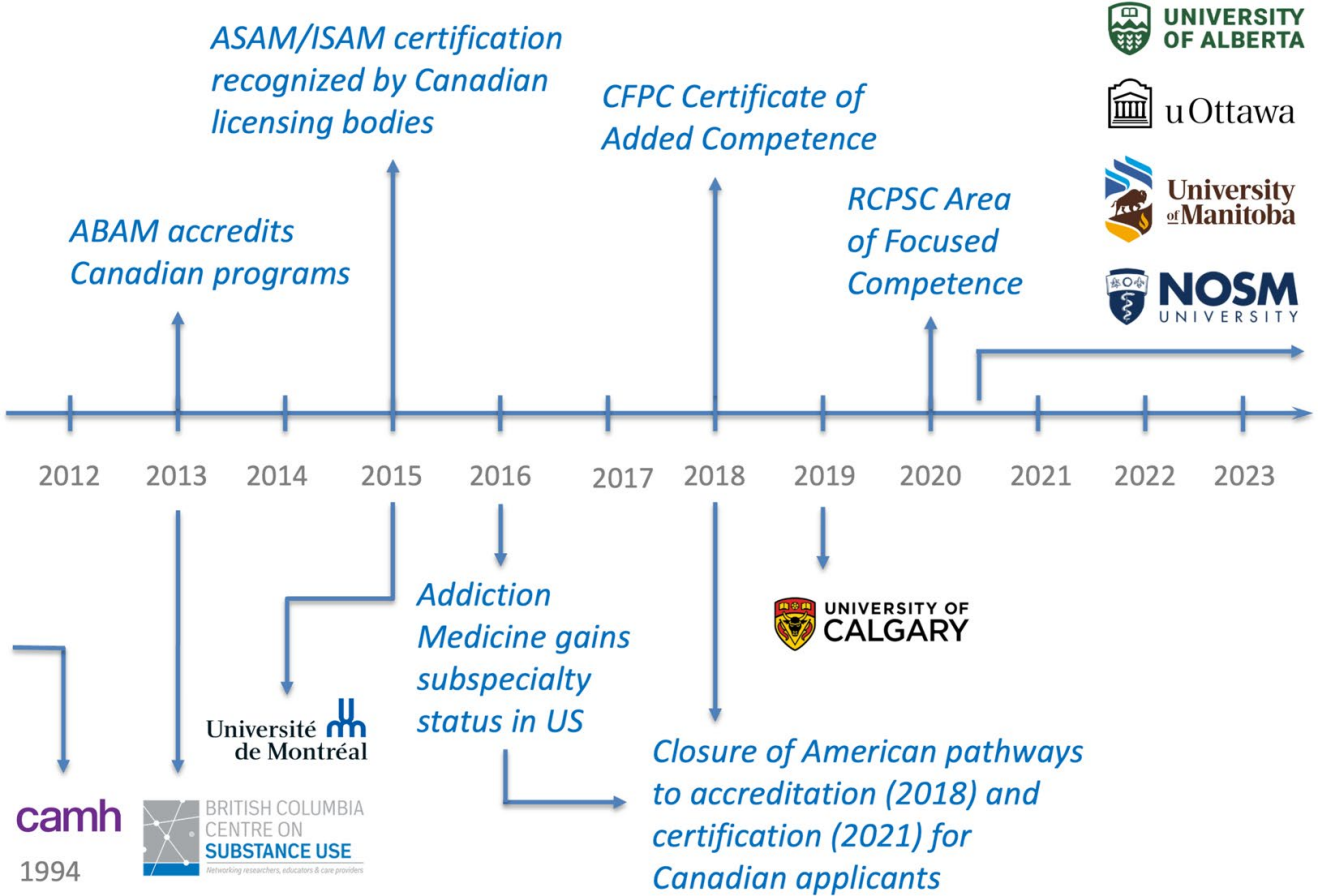
Evolution of Addiction Medicine fellowship training in Canada. *ABAM* American Board of Addiction Medicine, *ASAM* American Society of Addiction Medicine, *CAMH* Centre for Addiction and Mental Health, *CFPC* College of Family Physicians of Canada, *ISAM* International Society of Addiction medicine, *NOSM* Northern Ontario School of Medicine, *RCPSC* Royal College of Physicians and Surgeons of Canada, *uOttawa* University of Ottawa, *US* United States

In response to these calls, Addiction Medicine training opportunities in Canada have evolved substantially over the last decade [7, 8, 17–20], including the creation of two formal fellowship pathways. In 2018, the College of Family Physicians of Canada (CFPC) created a Certificate of Added Competence (CAC) in Addiction Medicine, while in 2020, the Royal College of Physicians and Surgeons of Canada (RCPSC) produced accreditation standards for an Area of Focused Competence (AFC) for non-family medicine trainees. Prior to these pathways, Addiction Medicine training standards in Canada trailed behind the United States (Fig. 1), with Canadian fellowships in Addiction Medicine relying on the American Board of Addiction Medicine for accreditation and trainee certification [8, 9].

The relative infancy of these formal training programs in Canada, combined with the pressing need for expert clinicians, creates challenges unique to the field of Addiction Medicine. First, clinical practice evolves rapidly, with knowledge dissemination and clinical guidance struggling to keep pace amid a limited evidence base, as evidenced by the wealth of interim guidance documents that emerged during the COVID-19 pandemic [21, 22]. More so than other fields, Addiction Medicine can be heavily influenced by stigma, political context, and personal ideology, making it challenging to teach [23–25]. Substance use disorders also manifest across all facets of medicine, requiring the involvement of multiple disciplines—by consequence, Addiction Medicine has no established “home” specialty to provide educational infrastructure and funding support.

Facing these unique challenges, Addiction Medicine education must be designed and scaled to address the enormous clinical need. However, there is scant literature on existing training programs at the national scale. Our research thus endeavours to characterize formal postgraduate Addiction Medicine training programs in Canada through a mixed methods qualitative study of Program Directors, allowing educators in this specialty to map the current state of training and navigate its future directions.

## Methods

### Research design overview

We conducted a mixed methods study of Program Directors (PDs) of Addiction Medicine clinical fellowship programs in Canada employing a sequential explanatory design. An initial quantitative survey (Phase 1) informed a subsequent qualitative interview (Phase 2) as illustrated in Additional file 1, Supplemental Fig. 1 [26]. We used the American Psychological Association Journal Article Reporting Standards to guide study reporting [27].

### Participant recruitment

All PDs of Canadian programs offering one-year clinical specialist training in Addiction Medicine were eligible and invited by e-mail to participate in both the survey and interview phases of this study. Directors of research-only fellowships, undergraduate medical education, and Continuing Medical Education activities were excluded.

### Researcher description

The research team comprised Addiction Medicine fellows trained in General Internal Medicine and Emergency Medicine, a medical residency PD with a background in Health Professions Education and experience in qualitative research, and a research scientist and Addiction Medicine Research Fellowship PD. Their combined experience informed this study’s quantitative and qualitative methodology and enriched its interpretation from the perspectives of both trainee and educator.

### Phase 1: quantitative survey

#### Survey development

We designed the survey tool with guidance from the Association for Medical Education in Europe (AMEE) Guide No. 87 on developing questionnaires for educational research [28]. This design process included literature review, expert validation, and involvement of previous PDs for cognitive interviewing and pilot testing of questions related to applicants, trainees, graduates, faculty and administrative support, curriculum, accreditation, scholarly activities, and program challenges during the 2021–22 and 2022–23 academic years.

#### Data collection

The survey was administered online in Spring 2023, with secure data collection and storage through the University of British Columbia (UBC) Survey Tool delivered by Qualtrics. We e-mailed eligible participants the survey link followed by reminder e-mails every two weeks until survey completion, decline of participation, or the end of the two-month survey period. The study was approved by our institutional Research Ethics Boards (REBs) and informed consent was collected at the onset of the survey.

#### Data analysis

We conducted an interim analysis of quantitative survey data using descriptive statistics. We reported frequencies and percentages on categorical variables and means and standard deviations on continuous variables.

### Phase 2: qualitative interview

#### Interview development

By examining quantitative trends during our interim analysis of survey data, we identified key themes informed by a literature review to explore in the semi-structured interview. We created a standardized interview guide probing: pathways to training, program funding, demand for graduates, standardization efforts, Equity, Diversity, and Inclusion (EDI), and future “moonshot” goals. We maintained flexibility toward integrating new themes through emergent research design and a semi-structured, iterative approach to interviewing participants.

#### Data collection

Two interviewers conducted virtual semi-structured interviews through the video conferencing platform Zoom. The interview was REB-approved and both written and verbal consent was obtained from study participants. Interviews were audio-recorded, transcribed, and de-identified. We strengthened our research processes and confirmed our qualitative findings through peer debriefing (with qualitative researchers and addiction medicine educators) and reflexivity (both interviewers recorded and discussed written memos reflecting on content and process after each interview). We strengthened credibility through investigator triangulation, expert validation, and member checking during and after interviews, with all participants provided the opportunity to review the deidentified transcript from their interview.

#### Data analysis

We analyzed interview transcripts using the qualitative data analysis software program Delve [29]. We used the thematic analysis approach outlined by Braun and Clarke [30] to inductively generate a coding scheme through a multi-layer tree structure. One researcher conducted three rounds of coding (CL): first, across individual transcripts to generate initial codes and collate potential themes; second, across coded extracts in relation to emerging themes; and third, across the entire data set to finalize themes, which were audited by another researcher (KC). We created a thematic “map” of the analysis to conceptualize and distill key themes and relationships into a cohesive narrative.

### Mixing procedures

“Mixing” of quantitative and qualitative data occurred during two procedures of the research design (Additional file 1, Supplemental Fig. 1). First, the interim analysis of quantitative data from the survey (Phase 1) revealed key themes that substantially informed the subsequent development of the qualitative interview (Phase 2). Second, the quantitative and qualitative datasets were integrated during the interpretation phase to elaborate upon the key themes identified during the interim analysis, enabling a rich characterization of the current state, key gaps, and future directions of Addiction Medicine training in Canada.

## Results

### Quantitative results

#### Demographics

Twelve of 13 eligible PDs (92.3%) completed the quantitative survey. Demographics and characteristics of participating PDs and fellowship programs are reported in Additional file 1, Supplemental Tables 1 and 2, respectively.

**Fig. 2:**
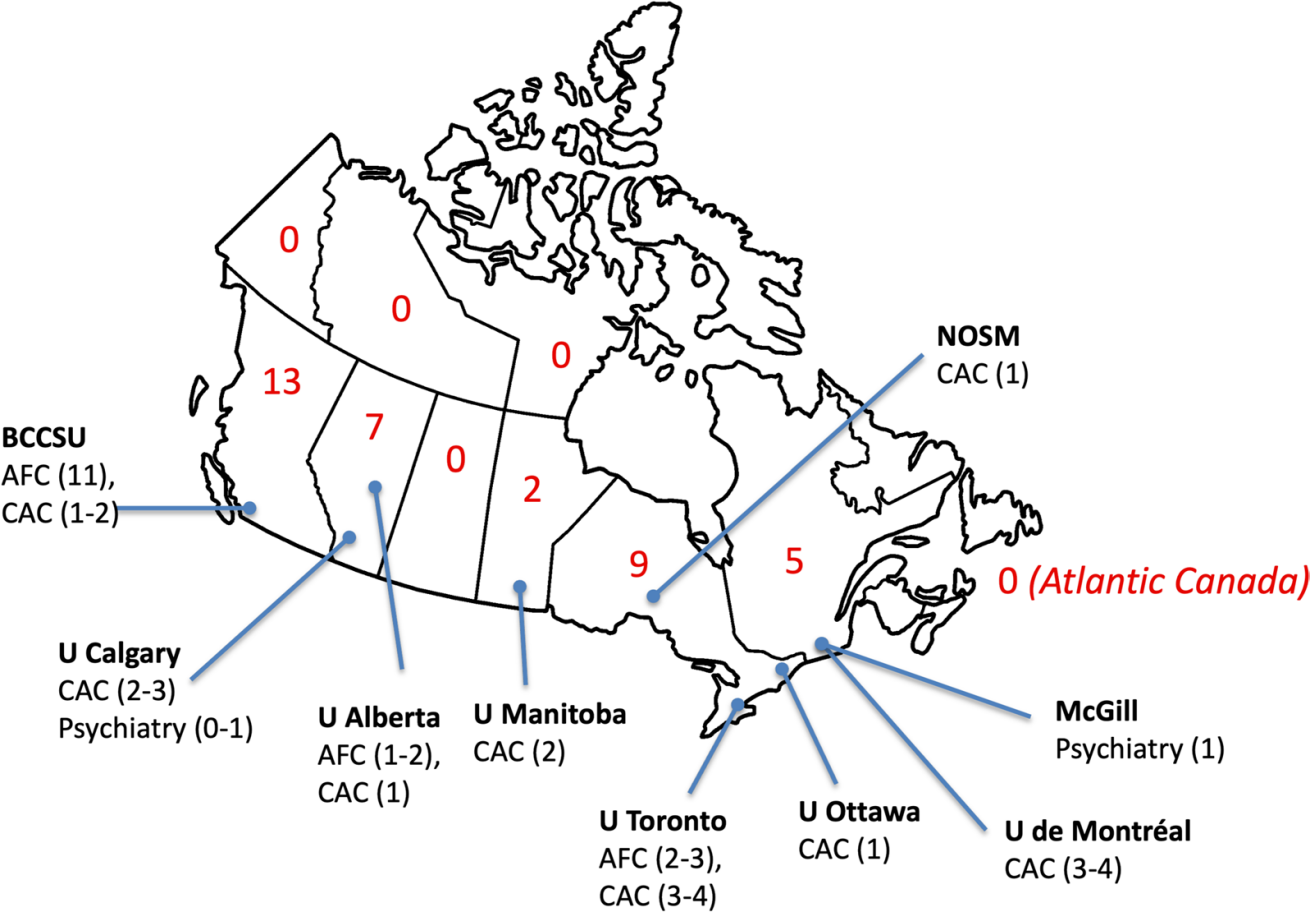
Geographic distribution of Addiction Medicine and Addiction Psychiatry fellowship programs and positions in Canada, 2021–2023. Red numbers indicate the number of available fellowship training positions available per province or territory. *AFC *Area of Focused Competence, *BCCSU* British Columbia Centre on Substance Use, *CAC *Certificate of Added Competence, *NOSM* Northern Ontario School of Medicine

The geographic distribution of available fellowship positions and programs, as reported by participants and/or publicly available information [31] is presented in Fig. 2. PDs were surveyed on the two most recent training cohorts at the time of survey administration, with demographics presented in Table 1. Trainees in Addiction Medicine programs originated from a wide variety of baseline specialities, with Family Medicine as the majority.

**Table 1:** Characteristics of Addiction Medicine Fellowship Trainees

	**2021–2022**	**2022–2023**
Application Cycle		
Number of Applicants	91	111
Available Spots	28 (30.8%)	32 (28.8%)
Number of Programs	10	11
Mean Applications per Program	9.1 (SD 4.16)	10.1 (SD 6.39)
Total Enrolled Trainees		
	28	27
Trainee Program		
CFPC—CAC	14 (50.0%)	14 (51.9%)
RCPSC—AFC	13 (46.4%)	12 (44.4%)
Addiction Psychiatry	1 (3.6%)	1 (3.7%)
Trainee Background		
Family Medicine	17 (60.7%)	18 (66.7%)
Psychiatry	6 (21.4%)	1 (3.7%)
Internal Medicine	4 (14.3%)	3 (11.1%)
Emergency Medicine	0	3 (11.1%)
Obstetrics and Gynecology	0	1 (3.7%)
Anesthesia	1 (3.6%)	0
Pediatrics	0	1 (3.7%)

*AFC* Area of Focused Competence, *CAC* Certificate of Added Competence, *CFPC* College of Family Physicians of Canada, *RCPSC* Royal College of Physicians and Surgeons of Canada

#### Program curriculum

Accreditation status of Addiction Medicine programs is presented in Additional File 1, Supplemental Table 3. All programs spanned 52 weeks, with a mix of mandatory and elective rotations. Additional file 1, Supplemental Fig. 2 displays the most common clinical rotations offered across all programs.

Trainees received an average of 3.33 (SD 1.97) formal academic sessions per month, including half days, journal clubs, and grand rounds. Academic characteristics of programs are summarized in Additional file 1, Supplemental Table 4. All programs offered the opportunity for interdisciplinary collaboration (through academic sessions, research activities, journal clubs, conferences, or social and wellness activities) with nursing, pharmacy, social work, research, and/or peer support colleagues.

#### Program administration

Five of 12 Addiction Medicine programs (41.7%) were based at an institution holding a Department or Division of Addiction Medicine. Program faculty size varied widely across programs (Additional file 1, Supplemental Table 4) and preceptors came from diverse backgrounds including family medicine, psychiatry, internal medicine, anesthesia, emergency medicine, pediatrics, obstetrics, and public health. Across programs, PDs estimated that 49% (SD 25.3%) of their preceptors underwent formal fellowship training themselves.

The average Full Time Equivalent (FTE) available to the PD was 0.15 (SD 0.09), where 1.0 FTE represents full-time work, and 50% of PDs spent more time than allocated in their stated role (Additional file 1, Supplemental Table 1). An average of 0.69 total FTE (SD 0.91) was available for program administrative staff.

#### Trainee funding

Trainees were funded through a variety of internal and external sources (Additional file 1, Supplemental Fig. 3). In some cases, trainees were partially funded through multiple sources.

#### Graduates

On average, PDs reported 97% (SD 9%) of graduates from the 2021–2022 cohort were working in Addiction Medicine. Thirteen of 27 graduates remained involved in the program for teaching or research. The majority of Program Directors perceived employment demand for graduates to be “very high” (90%).

#### Program challenges

PDs rated challenges to their Addiction Medicine training programs in terms of difficulty on a 7-point Likert scale, with ratings displayed in Additional file 1, Supplemental Fig. 4a. Assuring adequate funding for applicants was identified as the most difficult challenge. Program Directors were also asked to rank these same 12 challenges in terms of importance, from 1 = Most Important to 12 = Least Important, with results displayed in Additional file 1, Supplemental Fig. 4b. Funding to offer training to qualified applicants (M 3.75, SD 3.61) and faculty development, including having enough qualified teaching faculty (M 3.75, SD 2.13) were the most noted challenges.

### Qualitative findings

Ten Program Directors consented to participate in the qualitative interview. We synthesized key themes from their perspectives on the current state, medical education gaps, and future directions for Canadian education in addiction medicine.

#### Current state

##### Addiction medicine practice context

Participants identified several unique features of the existing Addiction Medicine landscape in Canada affecting the field’s capacity for medical education. First, Addiction Medicine is a relatively new field that continues to gain “increasing recognition” [P1] as a “legitimate” [P10] specialty—as one participant described, “Not just, ‘*Oh, that's a nice thing for you people to do*,’ but very much a necessity.” [P1].

Second, participants highlighted the interdisciplinary nature of practicing addiction medicine, which “adds a richness to our field” [P2] but also means that Addiction Medicine has not historically belonged to a single home specialty. This leads to an “orphan situation” [P5] with fragmentation of traditional funding models and clinical infrastructure.

Third, participants emphasized the “urgency” [P6] of scaling up the Addiction Medicine workforce “given how deep a hole we are in, trying to dig ourselves out … just to have enough people out there to meet the need at baseline.” [P6] Several alarm statements spoke to the urgency of addressing this care gap: “We need all hands on deck, quite frankly.” [P5].

Finally, participants described unique sociopolitical factors holding back an appropriate response to this Addiction Medicine care gap, including a tenuous political climate “that does not always support evidence-based addiction medicine,” [P10] “divides in addiction medicine” [P10] around controversial topics like safer supply, and longstanding stigma regarding substance use.

##### Addiction medicine education context

In parallel to this specialty forging its identity within the Canadian clinical context, addictions medical education has also experienced substantial evolution—particularly during the last decade’s establishment of Canadian accreditation pathways distinct from a previous reliance on American standards (Fig. 1). As one participant remarked, “We have our own processes that reflect our own unique needs and identity.” [P5]

Contributing to this evolution, five new Canadian fellowship programs launched and nine of 12 participants assumed the PD role since 2020. These participants spoke often on the theme of novelty and the significant learning curve and workload involved in building “from the ground up” [P1]; however, this novelty also made them “keen to collaborate.” [P10]

PDs felt that most physicians are “extremely ill-equipped” [P1] to manage Addiction Medicine, although it “affects all areas of medicine.” [P5] They identified a strong need for “demystifying addiction medicine” [P4] and providing generalists with “bread and butter” [P10] competencies earlier in undergraduate and core postgraduate medical training, acknowledging that “you don’t necessarily need to do a full year of training” [P9] to practice Addiction Medicine with acceptable competency.

As such, directors underlined the importance of training both generalists with a “baseline competency in management of … substance use disorders” [P6] and specialists who become “champions in those respective fields to advance this really important area of medicine … one that is still finding its way.” [P6] Rather than competing, specialists and generalists practicing Addiction Medicine were envisioned to have distinct yet reinforcing roles whereby specialists of diverse backgrounds mobilize and build capacity among generalists in their field. Within this framework, CFPC PDs highlighted the vital role of training family physicians to specialize and champion the integration of Addiction Medicine into the wraparound care delivered in primary care settings.

Meanwhile, RCPSC PDs felt that AFC programs for non-family medicine trainees are held back by a “complete lack of funding,” [P5] more stringent RCPSC accreditation standards, and unique administrative challenges related to an interdisciplinary training environment. However, as one PD highlighted, “the challenges are vastly outweighed” by the “enormous benefits to training people from a variety of backgrounds” [P6].

These extra barriers related to accreditation, administrative capacity, and funding appear to prevent CFPC PDs (who otherwise “would very much like to … train people from other disciplines” [P3]) from expanding to offer a parallel RCPSC program—or, in the grander scheme, merging CFPC and RCPSC training pathways.

Notably, some participants at institutions offering both CFPC and RCPSC fellowships reported that their sister programs are functionally “more or less identical” [P9] and “run fully integrated as opposed to two separate programs in parallel.” [P7] Some PDs looked to other specialties like Palliative Care, Emergency Medicine, and Anesthesia to better understand the potential for aligning CFPC and RCPSC training pathways. One director remarked, “I would have dearly loved if the RCPSC and the CFPC aligned … from the beginning … Now it feels like we've already gone far too far down the path to actually come back together … without one of us kind of giving way, which is a shame." [P6]

Once trainees graduate, PDs described a variety of job opportunities and practice models, with most graduates pursuing a blended clinical practice (either integrating or alternating Addiction Medicine with their background specialty) along with a notable minority practicing “solely as an addiction provider.” [P7]

Finally, PDs also emphasized the importance of training the next generation of Addiction Medicine specialists for roles beyond the medical expert (such as advocate, researcher, and leader), mirroring survey results that most programs require academic, research, and interdisciplinary opportunities beyond the clinical. PDs spoke about individualizing the preparation for these roles to fellows’ training background: “How do we create these experts who are going to … influence care for people who use substances, beyond the actual care they provide? I think that influences a great deal how we're thinking about trying to instill these values beyond clinical practice that are influenced by the person's training background." [P9]

#### Medical education gaps and future directions

##### Funding as the bottleneck

Mirroring our survey results, every interviewed Program Director underscored insufficient funding as the prevailing barrier and “perennial challenge” [P6] holding back the expansion of addictions training opportunities to meet a gaping clinical need.

**Fig. 3:**
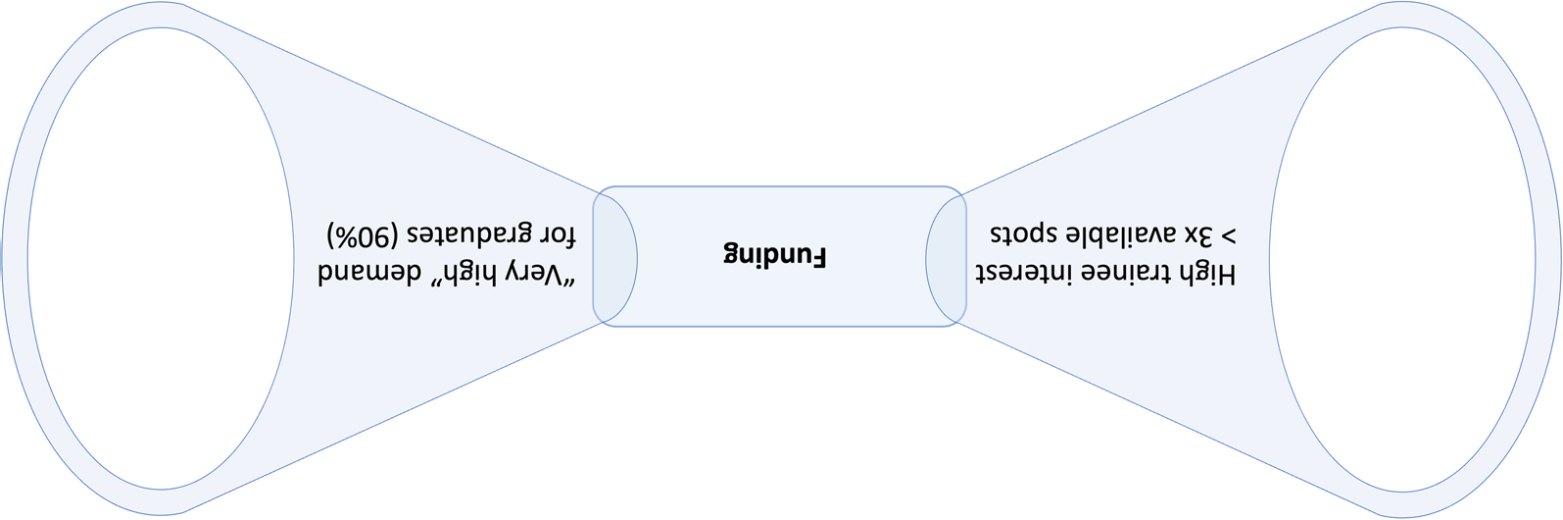
“Funding as the bottleneck” theme. Funding was described as the bottleneck between high trainee/applicant interest and very high perceived demand for graduates

Aligned with quantitative data (where fellowship positions were available to less than a third of applicants), PDs described both high trainee interest in obtaining Addiction Medicine education and high workforce demand for graduates of fellowship programs. However, they described a bottleneck effect (Fig. 3) where, despite the urgency of the opioid overdose crisis, available funding consistently fails to keep up with both the number of qualified applicants and demand for graduates of addiction medicine fellowships: “Often it feels like the restriction of how many people we end up taking is not dictated by applicant strength, but rather by the program size, which is limited more so by funding than training opportunities.” [P9] As a result, interested and capable practitioners are turned away from training and filling substantial care gaps.

Beyond the usual constraints of funding medical education, unique factors substantially limit access to funding for Addiction Medicine education. First, the field’s novelty has proven a barrier to accessing traditional, centralized postgraduate specialty funding as “all the universities are just figuring this out and are pretty apprehensive of taking on increased cost, especially in the long term.” [P6] Second, addiction medicine education does not intuitively belong to a single parent specialty or even regulatory college, creating an “orphan situation” [P5] with fragmented funding capacity. Lastly, Addiction Medicine education funding may be deprioritized across training levels due to deep-rooted stigma reducing substance use disorders to a “moral issue” [16] in contrast to other medical conditions. One participant described the response to the COVID-19 pandemic compared to the overdose crisis: “[COVID-19] crossed political lines. It was just like, this is a crisis, and we need to help. We all need to get working on this … That, I think, is what we need with the opioid crisis. But I think the stigma behind it is what’s changing the response level.” [P10]

Moving forward, PDs underscored that Addiction Medicine education funding must evolve toward more sufficient and sustainable models. This need was emphasized “particularly for the Royal College positions,” [P8] which receive no dedicated fellowship-specific funding compared to CFPC Enhanced Skills positions that are funded through Postgraduate Medical Education. The current potpourri of funding sources (Additional file 1, Supplemental Fig. 3) where programs without guaranteed funding “essentially look for money elsewhere [P9]” is tenuous, unsustainable, and “very much varies year to year [P9].” Of note, in the two-year survey period, no trainees were funded through private donors or foundations, though this had been a funding source historically [14].

PDs identified the Ministry of Health as the most appropriate source for delivering sustainable funding through a centralized, equitable model: “In an ideal world, I think it would be the government that was providing all the funding … and if there was a way to not differentiate between family medicine versus Royal College, or have some sort of equitable way of accessing that … they would make it better for the system.” [P8] To champion this increased funding from the Ministry, PDs called on “senior leadership” [P9] within universities, medical associations, and health authorities to “advocate strongly that—if there's capacity, and if this is an important training program—we need to have more rapid scale-up of funding that's available to us every year.” [P9].

##### Capacity versus burnout

The dearth of funding relative to need creates a medical education landscape ripe for burnout among leadership. Discussing PDs’ limited capacity and high turnover rates, one participant described the role as “overwhelming for a part-time position” [P4] while another shared, “I think we're all just tired … There's not very much funding for program directors, so this job is largely unpaid … It's difficult to recruit people for long-term when you're not paying them very well. And they're working really hard.” [P10]

Our survey indicated an average full time equivalent (FTE) of 0.15 allocated to PDs, amounting to less than one full day per week, and an average FTE of 0.69 for administrative support per program. While advocating for increased fellowship positions, PDs called for “a commensurate increase in program director time … Also, the administrative support would have to be kicked up a notch to support that.” [P2]

Beyond PD and administrative capacity, preceptor and clinical site capacity was also recognized as finite. Fellows may find themselves “contending” [P7] with elective learners for addictions rotations, with one PD describing the challenge of “wanting all the trainees to have a solid learning experience—but of course there are some who committed to doing a full year in this, so we have to ensure that their training needs are met.” [P7]

With a limited number of preceptors tied to each program, PDs ranked having enough qualified faculty as the second most important program challenge and were conscious of “not burning them out.” [P4] Addiction services for special populations (such as youth, perinatal addictions, and chronic pain) were seen as “the most challenging core areas” [P6] to provide sufficient exposure, along with rural and remote experiences.

A resounding theme, therefore, is that current clinical and educational demands on Addiction Medicine fellowships greatly outweigh capacity at the level of preceptors, clinical sites, and program administration and direction. As one PD remarked, “there’s just a massive amount of need and … so few addiction docs. So all of us are stretched thin all the time.” [P10]

##### The need for collaborative networks

PDs identified collaboration between programs as a powerful means of preserving capacity and mitigating burnout by sharing resources, avoiding redundancy, and creating a sense of both community and legitimacy as a specialty.

While programs were previously “fairly siloed” [P9] with individual, sporadic relationships between programs, PDs spoke optimistically about more recent efforts “connecting all of the fellowship directors … so that we can all work together instead of us all repeating and doing the same work” [P8] while recognizing “just how much we all have in common across the country.” [P5] Collaborations between programs have included fellowship program open houses and case rounds, with future plans for national career nights and a “shared drive where we can post our learning objectives and our rotations” [P8] with the intent to share unique elective experiences. PDs credited many of these early efforts to the Canadian Society of Addiction Medicine (CSAM) and the RCPSC.

PDs emphasized the need to further leverage a national, centralized collective: “Why not pool what's worked, and what hasn't, and have more of a network across the country?” [P7] Such a network could be organized to share educational resources “a bit more centrally … to reduce the burden and allow these smaller programs to operate” [P2] and “standardize… between provinces.” [P10] It could also bolster individual PDs building a new program or navigating accreditation for the first time: “When a new program is trying to come online, how can we share?” [P8] Potential international exchanges were also highlighted as an opportunity to share practice innovations that are well-established in parts of Canada, such as managed alcohol programs and injectable opioid agonist therapy: “I have an incredible amount of international requests to do training” to gain exposure to “some innovative things that they just don’t have access to in the States.” [P5]

##### Balancing structure and flexibility

The recent advent of fellowship program accreditation processes from both the CFPC and RCPSC introduced “national standards” [P5] that have been “helpful in legitimizing addiction medicine” and “advocating for funding for specific things” [P10] from senior leadership. Elaborating on the survey finding that accreditation was rated as more challenging by RCPSC than by CFPC PDs, participants described CFPC standards as more centralized and “vastly easier to meet,” [P6] with one wondering whether “the burden of the [RCPSC] accreditation piece does throw off some [CFPC] programs who might otherwise apply and be credentialed.” [P7]

Pervading PD interviews was the desire for even more flexibility in the way we imagine medical education infrastructure and funding models for Addiction Medicine: ““It should be appropriately flexible that you meet those requirements in whatever way fits your program best." [P9] For example, PDs “would like to see reduced barriers for people who are already in practice” [P2] and physicians new to practice who cannot afford another full year of training: “Especially if you're fairly early in your career, you're going to have financial obligations that the salary of a PGY3 is not going to meet.” [P1].

To expand access for physicians who cannot commit a full year at a time to addiction medicine training, PDs envisioned formalizing shorter periods of training to create a “middle ground” [P9] where physicians could “learn a lot in three months.” [P6] This could assist more physicians in eventually achieving certification through a “practice-eligible route” [P1] without completing a full fellowship. While limited funding exists at certain centres for family physicians to undergo CFPC “Category 2” Enhanced Skills training for up to six months, this funded option simply “doesn’t exist for the Royal College” [P6], with several PDs calling for “more formalization” [P6] and funding of such a pathway.

Ultimately, when “all hands on deck” [P5] are needed to address the urgent opioid crisis, PDs expressed that there should be multiple on-ramps to train as many qualified trainees as demonstrate interest. These alternative pathways require formalization and funding—along with more flexibility to redistribute existing educational and financial resources that overlap with, or go unused by, traditional pathways.

## Discussion

To our knowledge, this is the first study to characterize Addiction Medicine fellowship training at the national level in Canada, and the first in North America to use mixed methods to enrich quantitative data with the compelling voices of program leadership. Our work captures PDs’ perspectives on key gaps in addictions medical education and, importantly, summarizes their proposed future directions to address these gaps. In the face of exponential clinical need and rapidly evolving medical education standards, our findings will be relevant to educational institutions, policy writers, and leaders in addiction medicine education worldwide.

Data from Statistics Canada and quantitative results from our survey reveal that Canadian Addiction Medicine training programs added approximately 0.66–0.71 new addiction medicine physicians per year per 1 million people in Canada during the 2021–2023 period studied [32]. In our study, PDs highlighted several challenges in Canadian addictions education limiting its urgent expansion to meet training, workforce, and geographic needs. Near universally, PDs stressed that funding was the challenge of prime importance, echoing recent studies of American training programs [13, 15]. Funding is the rate-limiting step in ensuring programs have adequate capacity to support fellowship positions along with the vital educational infrastructure of preceptors, administrative support, and program leadership. Flexibility and collaboration were identified as two other areas for growth required to adapt to a continuously evolving landscape of addictions training and practice. Structures of accreditation are the educational scaffold from which—as a vine grows with tropism within its environment—there should be sufficient flexibility for alternative pathways to branch toward targeted areas of training needs in addiction medicine.

Rising to these challenges, PDs collectively offered several paths forward that may be applied to Canadian training programs and used as a model internationally, synthesized in Additional file 1, Supplemental Table 5. Once again, the most resounding of these underscores the need for robust funding support: first, to sustainably fund more Addiction Medicine fellowship positions; second, to increase postgraduate trainee access through alternative pathways (including non-fellowship routes); and third, to support the educational infrastructure required to train them.

PDs were also eager to see early program collaboration efforts evolve into a powerful national community of practice whose leadership could advocate for this funding, support individual programs in matters such as accreditation, and increase educational capacity across Canada and internationally. Commenting in 2014 on the need for strong national collaboration to advance addictions education and research, Hering et al. identified the CSAM, the Canadian Centre on Substance Use and Addiction (CCSA), and the Canadian Research Initiative in Substance Misuse (CRISM) as key organizations that could help lead the coordination and stewardship of a Canadian community of practice in Addiction Medicine education [7].

Our study also complements the work of Klimas et al. in 2017 exploring the clinical and research fellowship experiences of trainees and preceptors in Vancouver, Canada. This qualitative study examined the barriers and facilitators of implementing physician training in addiction medicine. Aligned with our current study of PDs across Canada, trainee and preceptor respondents in Vancouver spoke of the substantial impact of funding (after a large philanthropic gift in 2012) in empowering their fellowship program to build a strong educational infrastructure, along with the value of having a sturdy ‘backbone’ of faculty and staff capacity to support and mentor trainees [14]. In our study, PDs called for more robust funding models that could sustain programs nationally beyond a tenuous dependence on philanthropy.

Two previous surveys of Addiction Medicine fellowship PDs implemented in 2011 and 2020 informed the development of the first phase (quantitative survey) of our mixed methods study. Tontchev et al. conducted a survey of 14 Addiction Medicine and seven Addiction Psychiatry programs in the United States in 2011, describing heterogeneity in the range of clinical and research activities offered during these fellowships, along with available funding sources [15]. More recently, Derefinko et al. conducted a cross-sectional survey of 46 North American fellowship programs (43 in the US and 3 in Canada) with accreditation status in 2017 [13]. Parallel trends were seen in our study and this largely US-based study, in which most PDs (79.5%) also reported a high demand for graduates, and funding was widely rated as the most important need. This survey also described a broad array of funding sources, from hospital systems to corporate partners. Of note, both studies occurred before American programs gained access to funding for accredited fellowships in 2018, when Addiction Medicine was formally recognized as a certifiable specialty by the American Council for Graduate Medical Education (ACGME).

Since these studies, Canadian and American Addiction Medicine fellowship programs have diverged with the closure of American pathways of accreditation and certification to Canadian programs, and the development of independent Canadian accreditation standards by the CFPC and RCPSC. Most notably, Addiction Medicine and Addiction Psychiatry have forged entirely different training pathways in the US, while more convergence exists in Canada. Our qualitative analysis pointed to the rich potential for collaborations across Canada and internationally through clinical exchanges and medical education research, where trainee exposure to different clinical and educational innovations could inspire advances in local practice and policy landscapes. Indeed, several innovations that are well-established in leading Canadian centres (such as sustained-release oral morphine, injectable opioid agonist therapy, and managed alcohol programs) could inform research, education, and practice in other countries, and vice versa [33–38]. Our study further adds a Canadian lens to themes that will resonate worldwide, summarizing proposed future directions (Additional file 1, Supplemental Table 5) to address priorities of funding and capacity along with the challenges of building legitimacy and harmonizing education standards as a specialty (10, 39).

Strengths of our study include its careful attention to principles of mixed methods to richly elicit PDs’ perspectives on the current state and future aspirations for Addiction Medicine education in Canada. This work is timely in that it captures the juncture of dual public health crises (the overdose epidemic and the COVID-19 pandemic), a season of controversy regarding best practices within the Addiction Medicine community and among political spheres, and the recent evolution of Canadian accreditation standards for addiction medicine. It further gives voice to PDs’ balance of cautious optimism and conviction in preparing future addictions providers to move these issues forward.

Limitations include this study’s focus on the perspectives of Addiction Medicine Program Directors in Canada—which likely overlap with but may not be fully transferable to other countries and stakeholders such as trainees, graduates, and preceptors. All eligible PDs were invited to this study, yielding a small sample size simply due to a limited number of programs across the country—still, a high response rate and diversity of perspectives were elicited. As this was a cross-sectional study, our quantitative results are limited to two consecutive years of training that may not speak to more longitudinal perspectives from program leadership. Finally, the timing of the study period during the peak of the COVID-19 pandemic may have influenced PDs’ perceptions and prioritization of their trainees’ educational needs; nonetheless, we believe that insights from this period can valuably inform post-pandemic efforts in medical education.

## Conclusion

In our mixed methods study of Addiction Medicine fellowship programs in Canada, PDs voiced that delivering training commensurate to the staggering clinical need requires sustainably increased funding, program collaboration, and a robust educational infrastructure balanced with sufficient flexibility to adapt to evolving trainee needs. To build momentum on these goals, Addiction Medicine leaders in Canada and worldwide must galvanize medical educators into an effective community of practice to train the next generation of addiction medicine providers. Future studies should examine the perspectives of Addiction Medicine fellows, the clinical and research impacts of graduates, and the cost-effectiveness of fellowship training models.

## Supplementary Information

Supplementary Material 1. Supplemental Fig. 1. Visual model for mixed-methods sequential explanatory design. Supplemental Table 1. Demographics of Addiction Medicine fellowship program directors. Supplemental Table 2. Characteristics of Addiction Medicine fellowship programs. Supplemental Table 3. Accreditation status of Addiction Medicine Training Programs. Supplemental Fig. 2. Most Common Rotations across Addiction Medicine Training Programs. Supplemental Table 4. Academic characteristics of Addiction Medicine Training Programs. Supplemental Fig. 3. Funding sources for Addiction Medicine fellows. Supplemental Fig. 4. Challenges to Addiction Medicine Training Programs. Supplemental Table 5. Future directions to address Addiction Medicine education gaps in Canada

## Data Availability

Additional data can be found in Additional file 1.pdf as above. The full datasets generated and/or analysed during the current study are not publicly available due to confidentiality restrictions as outlined in our REB agreements.

## Abbreviations

ACGME: American Council for Graduate Medical Education
AFC: Area of Focused Competence
CAC: Certificate of Added Competence
CFPC: College of Family Physicians of Canada
CCSA: Canadian Centre on Substance Use and Addiction
CRISM: Canadian Research Initiative on Substance Misuse
CSAM: Canadian Society of Addiction Medicine
EDI: Equity: Diversity: and Inclusion
FTE: Full Time Equivalent
M: Mean
RCPSC: Royal College of Physicians and Surgeons of Canada
PD: Program director
PGY: Postgraduate year
REB: Research ethics board
SD: Standard deviation
UBC: University of British Columbia
US: United States
