# Supplementary material for: “We need all hands on deck”: characterizing addiction medicine training in Canada—a mixed methods study of fellowship program directors
Source: Addict Sci Clin Pract. 2025 Feb 19;20:18. doi: 10.1186/s13722-025-00543-4 (PMC11837306; doi:10.1186/s13722-025-00543-4)
Supplement: Supplementary file 1 — Supplementary Material 1. Supplemental Fig. 1. Visual model for mixed-methods sequential explanatory design. Supplemental Table 1. Demographics of Addiction Medicine fellowship program directors. Supplemental Table 2. Characteristics of Addiction Medicine fellowship programs. Supplemental Table 3. Accreditation status of Addiction Medicine Training Programs. Supplemental Fig. 2. Most Common Rotations across Addiction Medicine Training Programs. Supplemental Table 4. Academic characteristics of Addiction Medicine Training Programs. Supplemental Fig. 3. Funding sources for Addiction Medicine fellows. Supplemental Fig. 4. Challenges to Addiction Medicine Training Programs. Supplemental Table 5. Future directions to address Addiction Medicine education gaps in Canada [file 13722_2025_543_MOESM1_ESM.pdf]

Additional File 1

Characterizing Addiction Medicine Training in Canada – A Mixed Methods Study of Fellowship Program Directors

Clara Lu, MD; Kathryn Chan, MD; Leslie Martin, MD, MHPE; Nadia Fairbairn, MD, MHSc

Table of Contents

Supplemental Figure 1. Visual model for mixed-methods sequential explanatory design p2
Supplemental Table 1. Demographics of Addiction Medicine fellowship program directors p3
Supplemental Table 2. Characteristics of Addiction Medicine fellowship programs p4
Supplemental Table 3. Accreditation status of Addiction Medicine Training Programs p6
Supplemental Figure 2. Most Common Rotations across Addiction Medicine Training Programs p7
Supplemental Table 4. Academic characteristics of Addiction Medicine Training Programs p8
Supplemental Figure 3. Funding sources for Addiction Medicine fellows p9
Supplemental Figure 4a. Challenges to Addiction Medicine Training Programs, ranked by Difficulty p10
Supplemental Figure 4b. Challenges to Addiction Medicine Training Programs, ranked by Importance p11
Supplemental Table 5. Future directions to address Addiction Medicine education gaps in Canada p12

Supplemental Figure 1. Visual model for mixed-methods sequential explanatory design

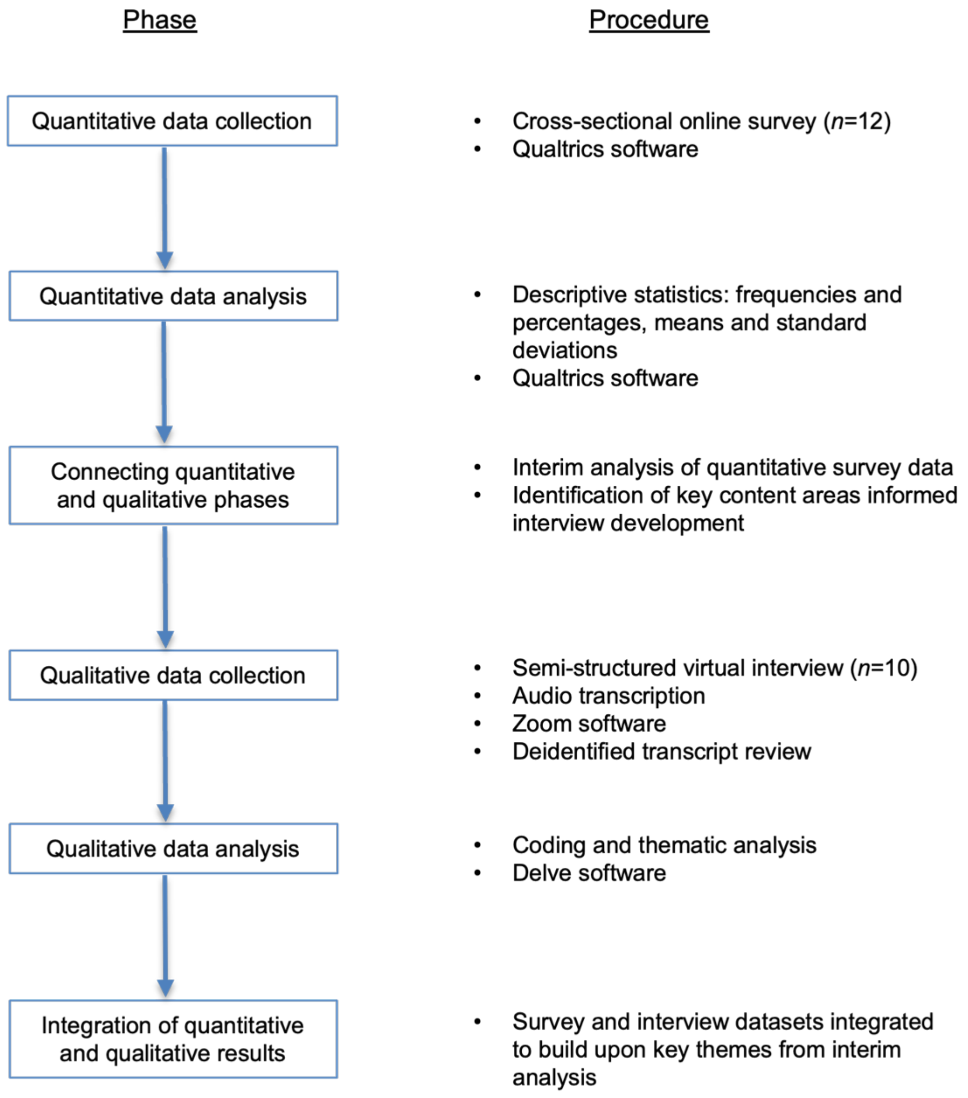

Supplemental Table 1. Demographics of Addiction Medicine fellowship Program Directors

	**Number of PDs**<br>*n* = 12
**Training Background of PDs**	
Family Medicine	8 (66.7%)
Psychiatry	3 (25.0%)
Internal Medicine	1 (8.3%)
**Mean Full Time Equivalent (FTE) per PD**	0.15 (SD 0.09)
PDs spending more than allocated FTE in their role	6 (50%)
PDs spending equivalent to allocated FTE in their role	5 (41.7%)
Not applicable	1 (8.3%)

FTE, Full Time Equivalent. PDs, program directors. SD, standard deviation.

Supplemental Table 2. Characteristics of Addiction Medicine fellowship programs

Institution	Location	Program Type	Eligibility	Year Established	Number of available fellowship positions per year (2021-2023 data)
BC Centre on Substance Use	Vancouver	RCPSC - AFC	All medical specialties<br>Applicants can be either in practice currently or in their last two years of any specialty residency training program	2013	11
BC Centre on Substance Use	Vancouver	CFPC - CAC	Final year resident trainees in Family Medicine	2013	1-2
University of Calgary	Calgary	CFPC - CAC	Final year resident trainees in Family Medicine	2019	2-3
University of Calgary	Calgary	Addiction Psychiatry Fellowship	RCPSC Psychiatry graduates	1998	0-1
University of Alberta	Edmonton	RCPSC - AFC	All medical specialities	2021	1-2
University of Alberta	Edmonton	CFPC - CAC	Final year resident trainees in Family Medicine	2022	1
University of Manitoba	Winnipeg	CFPC - CAC	Final year resident trainees in Family Medicine<br>Program is open to training physicians from other disciplines,	2020	2

			but they must bring their own funding		
Northern Ontario School of Medicine	Thunder Bay	CFPC - CAC	Final year resident trainees in Family Medicine	2023	1
University of Toronto	Toronto	RCPSC - AFC	All medical specialities	1994	2-3
University of Toronto	Toronto	CFPC - CAC	Final year resident trainees in Family Medicine	1994	3-4
University of Ottawa	Ottawa	CFPC - CAC	Final year resident trainees in Family Medicine	2021	1
Université de Montréal	Montréal	CFPC - CAC	Final year resident trainees in Family Medicine, French language program	2015	3-4
McGill University	Montréal	Addiction Psychiatry Fellowship	RCPSC Psychiatry graduates	Unknown	Unknown

AFC, Area of Focused Competence. CAC, Certificate of Added Competence. CFPC, College of Family Physicians of Canada. RCPSC, Royal College of Physicians and Surgeons of Canada.

** Denotes year of program establishment; note that data were not available for year of accreditation.*

Supplemental Table 3. Accreditation Status of Addiction Medicine Programs

	**CAC Programs**<br>*n* = 8	**AFC Programs**<br>*n* = 3
Fully accredited programs	7 (87.5%)	3 (100%)
Difficulty* of acquiring accreditation	3.88 (SD 1.27)	4.0 (SD 1.22)
Difficulty* of maintaining accreditation	3.88 (SD 1.36)	4.6 (SD 0.49)
Graduation requirement		
CAC certification	6 (75%)	N/A
AFC certification	N/A	2 (66.7%)
CSAM certification	0	0
ISAM certification	0	0

*Difficulty ranked on a 7-point Likert scale (1 ‘not difficult at all’ to 7 ‘extremely difficult’).

AFC, Area of Focused Competence. CAC, Certificate of Added Competence. CSAM, Canadian Society of Addiction Medicine. ISAM, International Society of Addiction Medicine.

Supplemental Figure 2. Most Common Rotations across Addiction Medicine Training Programs

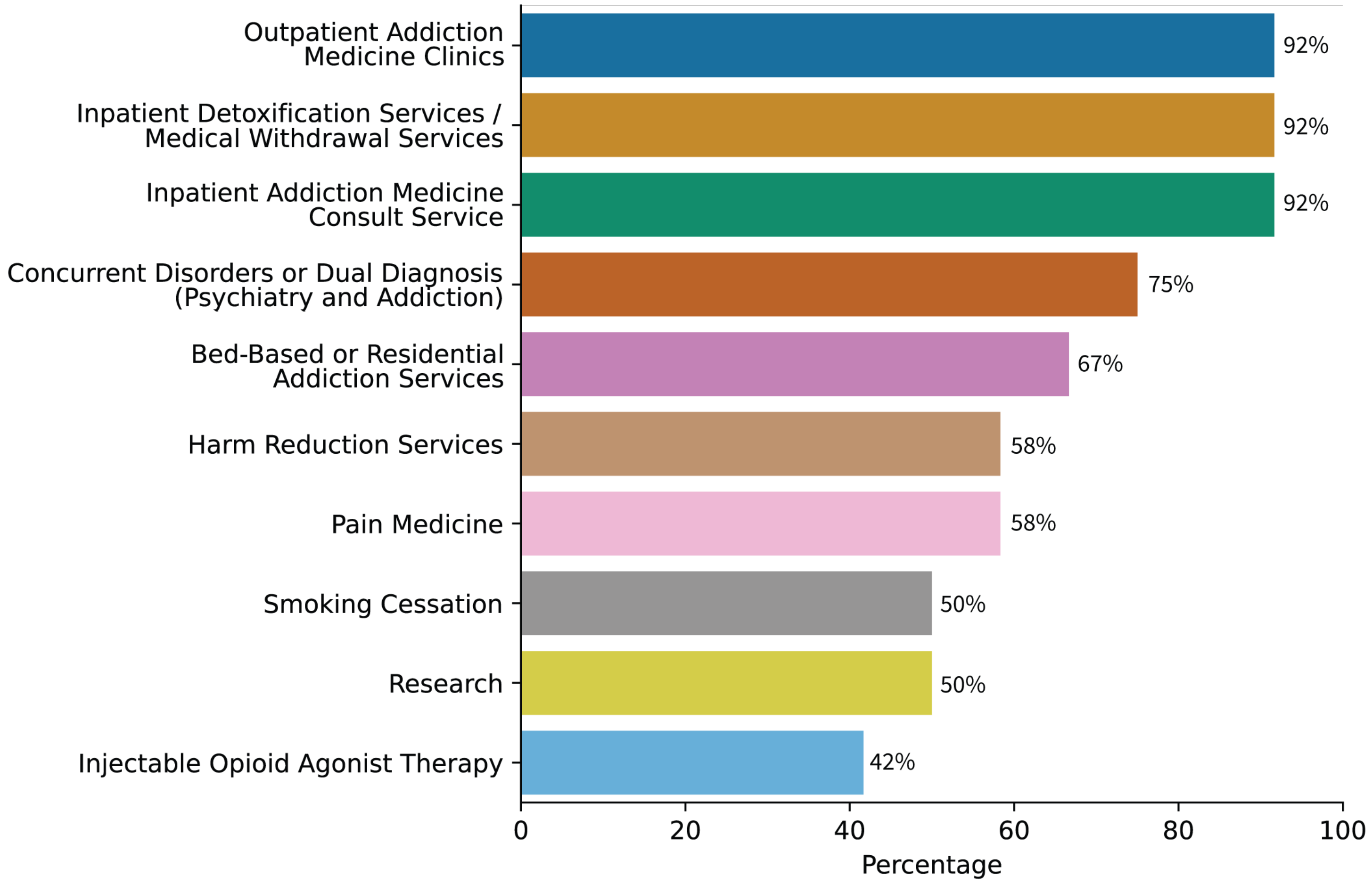

Supplemental Table 4. Academic Characteristics of Addiction Medicine Training Programs

	**Number of Programs** *n = 12*
**Academic Requirements**	
Addiction Medicine trainees required to supervise junior learners	10 (83.3%)
Addiction Medicine trainees required to present academic sessions	8 (66.7%)
Addiction Medicine trainees required to complete a research project	9 (75.0%)
**Trainee Evaluation**	
Competency Based Medical Education (CBME)	
Fully implemented	6 (50.0%)
Partially implemented	4 (33.3%)
Program has plans to implement	1 (8.3%)
Program has no plans to implement	1 (8.3%)
Methods of Assessment	
Entrustable Professional Activities (EPAs)	6 (50%)
In-training evaluations	11 (91.7%)
Additional assessment of scholarship activity	8 (66.7%)
Informal feedback	10 (83.3%)
Interdisciplinary evaluations	9 (75.0%)
**Program Faculty Size**	
0-5 preceptors	1 (8.3%)
6-10 preceptors	3 (25.0%)
11-15 preceptors	4 (33.3%)
16-20 preceptors	0
>20 preceptors	4 (33.3%)

Supplemental Figure 3. Funding sources for Addiction Medicine fellows

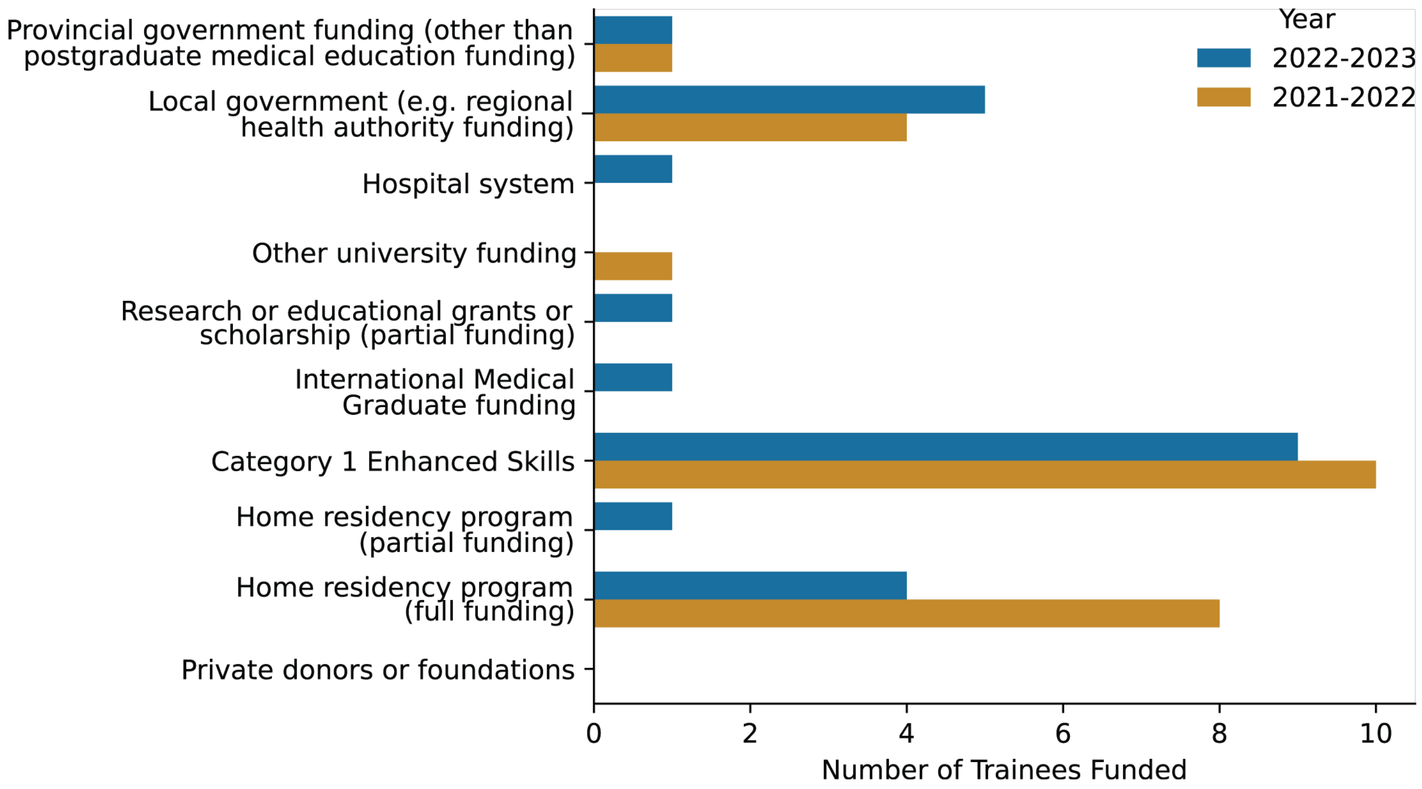

Supplemental Figure 4a. Challenges to Addiction Medicine Training Programs, ranked by Difficulty

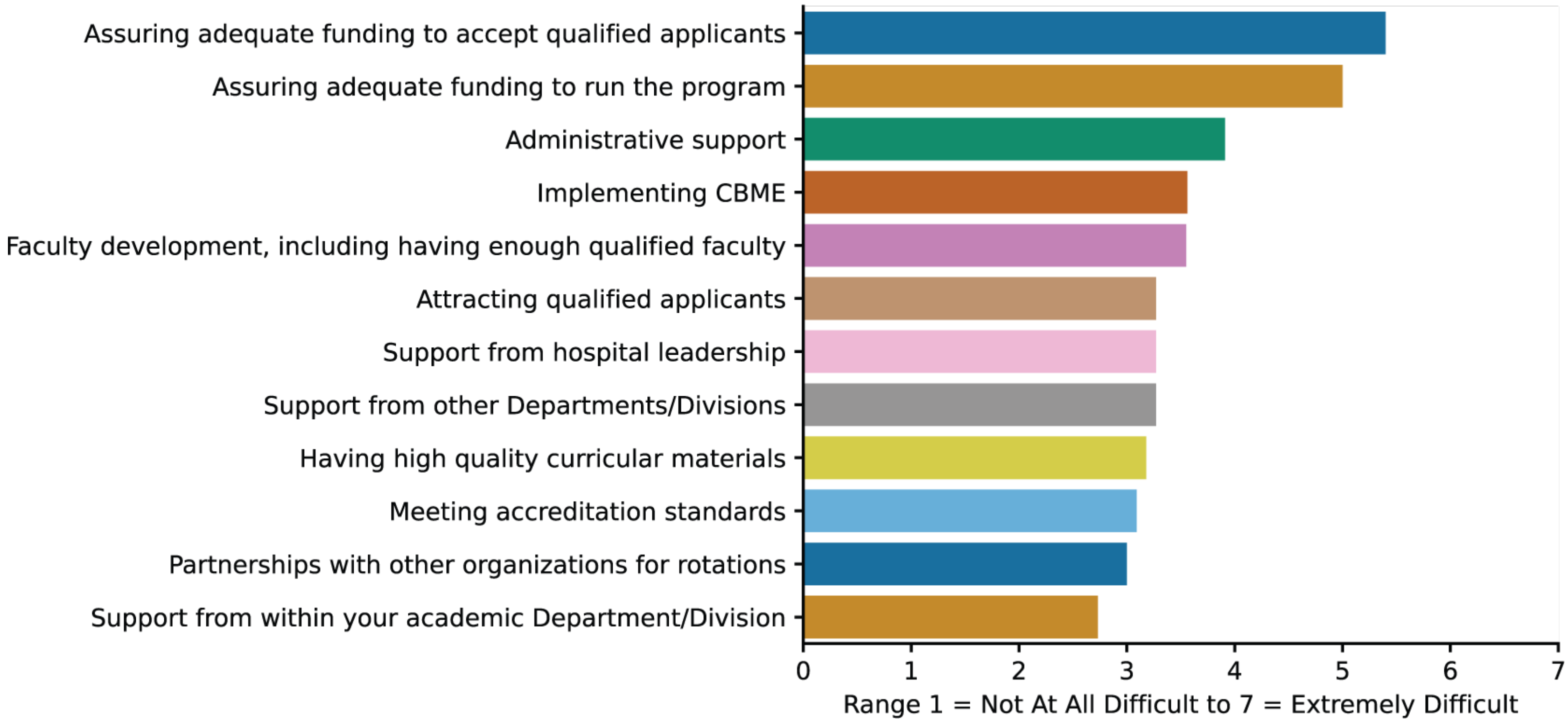

Supplemental Figure 4b. Challenges to Addiction Medicine Training Programs, ranked by Importance

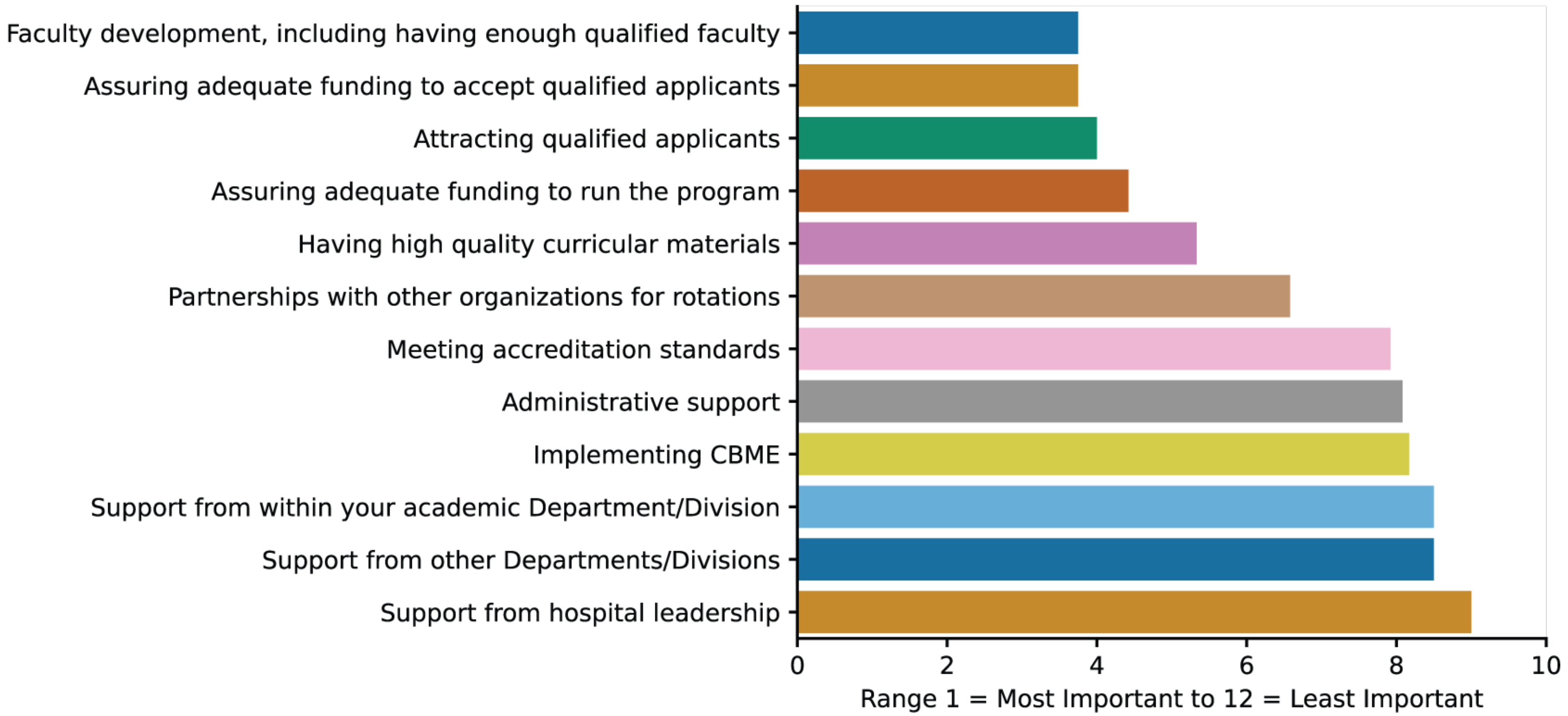

Supplemental Table 5. Future directions to address Addiction Medicine education gaps in Canada

Medical education gaps	Future directions
A. Funding as the bottleneck	1. Create dedicated and sustainable funding models for both RCPSC and CFPC Addiction Medicine trainees through a centralized source (such as provincial Ministries of Health), as championed by university and medical association leadership.
B. Capacity versus burnout	1. Secure additional funding to increase the capacity of preceptors, clinical sites, program directors, and administrative assistants to increase physicians' access to addiction medicine training.<br><br>2. Increase collaboration between fellowship programs to share resources, reduce redundancy, preserve capacity, and mitigate burnout.
C. Need for collaborative networks	1. Develop leadership and stewardship infrastructure to coordinate centralized efforts toward a national community of practice in Addiction Medicine education. These could draw from American models such as the American College of Addiction Medicine (ACAAM) and the Substance Abuse and Mental Health Administration (SAMHSA) Provider's Clinical Support System.
D. Balancing structure and flexibility	1. Produce centralized guidance (with flexibility in application) to reduce barriers in acquiring and maintaining accreditation.<br><br>2. Formalize and fund alternative postgraduate training pathways in Addiction Medicine (shorter durations, re-entry from practice), expanding flexibility in the redistribution of existing and unused funding for traditional pathways.

CFPC, College of Family Physicians of Canada. RCPSC, Royal College of Physicians and Surgeons of Canada.
